# The Transcription Factor Runx2 Is under Circadian Control in the Suprachiasmatic Nucleus and Functions in the Control of Rhythmic Behavior

**DOI:** 10.1371/journal.pone.0054317

**Published:** 2013-01-25

**Authors:** Meghan E. Reale, Ian C. Webb, Xu Wang, Ricardo M. Baltazar, Lique M. Coolen, Michael N. Lehman

**Affiliations:** 1 Department of Anatomy and Cell Biology, University of Western Ontario, London, Ontario, Canada; 2 Department of Neurobiology and Anatomical Sciences, University of Mississippi Medical Center, Jackson, Mississippi, United States of America; Florida State University, United States of America

## Abstract

Runx2, a member of the family of runt-related transcription factors, is rhythmically expressed in bone and may be involved in circadian rhythms in bone homeostasis and osteogenesis. Runx2 is also expressed in the brain, but its function is unknown. We tested the hypothesis that in the brain, Runx2 may interact with clock-controlled genes to regulate circadian rhythms in behavior. First, we demonstrated diurnal and circadian rhythms in the expression of Runx2 in the mouse brain. Expression of Runx2 mRNA and protein mirrored that of the core clock genes, *Period1* and *Period2*, in the suprachiasmatic nucleus (SCN), the paraventricular nucleus and the olfactory bulb. The rhythm of *Runx2* expression was eliminated in the SCN of *Bmal1^−/−^* mice. Moreover, by crossbreeding *mPer2^Luc^* mice with *Runx2^+/−^* mice and recording bioluminescence rhythms, a significant lengthening of the period of rhythms was detected in cultured SCN of *Runx2^−/−^* animals compared to either *Runx2^+/−^* or *Runx2^+/+^* mice. Behavioral analyses of Runx2 mutant mice revealed that *Runx2^+/−^* animals displayed a significantly lengthened free-running period of running wheel activity compared to *Runx2^+/+^* littermates. Taken together, these findings provide evidence for clock gene-mediated rhythmic expression of *Runx2*, and its functional role in regulating circadian period at the level of the SCN and behavior.

## Introduction

The mammalian timekeeping system that drives rhythmic processes occurring on a 24 hour cycle is organized by a hierarchy of oscillators whose phase is coordinated by the suprachiasmatic nucleus of the hypothalamus (SCN) [1], [2]. In individual SCN cells, as in peripheral cellular oscillators, circadian rhythms are driven by networks of transcriptional-translational auto-regulatory loops (TTL) having a cycle lasting approximately 24 hours. The basic helix-loop-helix transcription factor Bmal1 (brain and muscle ARNT-like 1) and Clock (circadian locomotor output cycle kaput) heterodimerize and drive transcription of core clock genes *Period* (*Per1*, *Per2* and *Per3*) and *Cryptochrome* (*Cry1* and *Cry2*) genes by binding E-box elements within gene sequences [3]. Per and Cry proteins accumulate in the cytoplasm where they form heteromultimeric complexes with other proteins and translocate into the nucleus to inhibit the activity of Clock/Bmal1. As the levels of *Per* and *Cry* expression and protein concentration decrease, the inhibitory complexes are degraded thus restarting the cycle. In addition to core clock factors, genes involved in basic cellular events are also tightly regulated by the TTL (for examples, see [4]–[7]). By regulating the rhythmic expression of these clock-controlled genes (CCGs), the circadian system coordinates rhythmic physiological processes at both cellular and tissue levels.

Runt-related transcription factor 2 (Runx2) is one of three members (Runx1–3) of the mammalian family of transcription factors harboring a runt domain, a highly conserved DNA-binding sequence first described in *Drosophila melanogaster* [8]–[10]. Recent studies point to a putative interaction between Runx2 and the circadian clock network in osteogenesis and bone homeostasis [11], [12]: a) clock gene mutant mice show deregulated bone formation parameters largely attributed to increased numbers of osteoblasts, a cell type whose development from its precursors is highly dependent on Runx2-mediated events [13], [14]; b) Runx2 expression and several of its downstream gene targets are rhythmically expressed in bone [15]; and c) oscillations in molecular clock genes are detectible in bone and in cultured osteoblasts [14], [16]. Since *Runx2* expression is also detectable in non-osseous tissues including pituitary, gonads and the brain [17]–[19], these findings suggest that *Runx2* may be a CCG in multiple tissues.

In this study, we first examined temporal expression of Runx2 mRNA and protein in the SCN and other brain regions known to be rhythmic, and compared its circadian profile to that of the core clock genes *Per1*, *Per2* and *Bmal1*. Second, we determined whether Runx2 was under control of the circadian clock by examining its expression in the SCN of *Bmal1*-null mice. Third, we compared molecular rhythms between SCNs cultured from *Runx2^+/+^*, *Runx2^+/−^* and *Runx2^−/−^* mice in real-time by crossbreeding *Runx2^+/-^* mice with mice harboring the circadian reporter gene construct, *mPeriod2::Luciferase* (*mPer2^Luc^*). Finally, we examined activity rhythms in *Runx2^+/−^* mice to determine whether Runx2 has a role in regulating behavioral clock outputs. Taken together, the findings from these experiments provide insight to the relationship between Runx2 and the circadian system including evidence for clock gene-mediated rhythmic expression of *Runx2*, and the functional role for Runx2 in regulating circadian period at the level of the SCN and behavior.

## Materials and Methods

### Animals

All animal care and experimental procedures were approved by the Canadian Council of Animal Care and the Animal Use Subcommittee of the University of Western Ontario.

All mice strains, including mutants, were generated on a C57BL/6 background. Adult, male C57BL/6 mice (8–10 weeks old; Charles River Laboratories, Montreal, Canada) were used for gene and protein expression studies. Male *Bmal1* knockout mice (*Bmal1^−/−^*) mice were generated by breeding *Bmal1^+/−^* mice (founder mice generously provided by Dr. C. Bradfield, University of Wisconsin Medical School, Madison, WI) [20]. Offspring genotyping was performed similar to the methodology previously described [20]. However, a non-multiplex PCR approach was used in place by of multiplex PCR since the mutant allele is expressed at lower levels than the wild type allele, which can lead to competitive primer binding during multiplex PCR resulting in instances of false negative genotyping results. Primers used for genotyping PCR: OL2646, 5′-CCACCAAGCCCAGCAACTCA-3′; OL2647, 5′-ATTCGG-CCCCCTATCTTC-TGC-3′; and OL278, 5′-TCGCCTTCTATCGCCTTCTTGACG-3′. The wild-type allele is amplified by primers OL2647 and OL2646 (WT primer set), which produce a 400 bp PCR product. The gene knock-in product (600 bp) is amplified by the neomycin-specific primer set OL278 and OL2646 (M primer set) since the region targeted by OL2647 is deleted from the mutant *Bmal1* gene. In brief, PCR reactions for each animal were performed using genomic DNA isolated from tail biopsies with 2× PCR buffer (10 µl; Promega) supplemented with 2.5 mM MgCl_2_ and either WT or M primer sets (1 mM of each primer). PCR reaction conditions consisted of 40 cycles of 95°C for 15 s; 60°C for 15 s; 72°C for 1 min. PCR products were separated by electrophoresis on a 1% agarose gel in 1× TAE buffer [40 mM Tris; 19 mM Acetic Acid (EMD Biosciences, San Diego, CA)]. Two PCR reactions were performed for each animal, one for each primer set, to determine which alleles were present.

*Runx2* deficient (*Runx2^+/−^*) mice were generated by breeding *Runx2^+/−^* mice (founder mice generously provided by Dr. S. Mundlos, Max Planck Institute for Molecular Genetics, Berlin, Germany) [21]. Offspring genotyping was conducted by PCR using the following primers (sequences provided by Dr. S. Mundlos): Neo5-neu2, 5′-GATGATCTGGACGAAGAGCATC AGG-3′; Cbfa11-neu, 5′-GGGTGACCAGTCTCTTACCTTG-3′; MCBFA3-korr, 5′-AGCGAC GTGAG-CCCGGTG -3′. In brief, multiplex PCR reactions for each animal were performed using genomic DNA isolated from tail biopsies with 2× PCR buffer (10 µl; Promega) supplemented with 2.5 mM MgCl_2_ and 2.5 mM of each primer. The wild-type allele produced a 350 bp PCR product while the mutated Runx2 (neomycin knock-in) gene had a 600 bp amplification product. PCR reaction conditions consisted of 35 cycles of 94°C for 1 min; 59°C for 1 min; 72°C for 1 min. For both transgenic strains, WT (*Bmal1* ^+/+^, *Runx2^+/+^*) littermates were used for experimental control groups.

Mice homozygous for the *mPeriod2::Luciferase* fusion protein gene (*mPer2^Luc^*; Jackson Laboratory, Bar Harbor, ME) were crossbred with *Runx2^+/−^* mice to generate *mPer2^Luc^*/*Runx2^+/−^* mice for *in vitro* bioluminescence recording experiments. Offspring genotyping was conducted for *mPer2^Luc^* as previously described [22]. In brief, multiplex PCR reactions were performed using 250 ng of genomic DNA isolated from tail biopsies with 2× PCR buffer (10 µl; Promega) supplemented with 2.5 mM MgCl_2_ and 2.5 mM of each primer. Primers used for genotyping PCR: P1, 5′-CTGTGTTTACTGCGAGAGT-3′; P2, 5′-GGGTCC-ATGTGATTAGAAAC-3′; P3, 5′-TAAAACCGGGAGGTAGATGAGA-3′. The wild-type allele is amplified by primers P1 and P2, which produce a 230 bp PCR product. The luciferase knock-in allele PCR product (680 bp) is amplified by primers P1 and P3. PCR reaction conditions consisted of 35 cycles of 95°C for 45 s; 55°C for 45 s; 72°C for 1 min. Genotyping for Runx2 was also performed, as described above. Crossbreeding *Runx2^+/−^/mPer2^Luc+/+^* produced litters with predicted Mendelian ratios for the three possible genotypes: *Runx2^+/+^*, *Runx2^+/−^* and *Runx2^−/−^*. Brain and liver were cultured in random order without prior knowledge of *Runx2* genotype; overall, viability of tissues in culture was similar regardless of genotype and did not correlate with the order in which tissues were collected from the uterine sac.

Transgenic breeding colonies were group housed and kept under a 12 h∶ 12 h light∶ dark (LD) cycle until experimental manipulations were conducted. During experimentation, animals were individually housed at constant room temperature and humidity in cages equipped with running wheels (MiniMitter, Bend, OR) and food and water available *ad libitum*. Running wheel activity was monitored and recorded continuously in 5 min intervals using VitalView software (MiniMitter). Actograms, periodograms and activity levels were analyzed using Clocklab software (Actimetrics, Evanston, IL).

For studies of diurnal and circadian rhythms in gene and protein expression, animals were housed in LD until running wheel activity was observed to be stably entrained (10–14 days) and subsequently either maintained in LD (7–10 days) or housed under constant dim red illumination (lux<5; DD) until stable free-running behavior was established (10–14 days). For animals housed in LD, by convention, lights ON is designated as zeitgeber time 0 (ZT0) and lights OFF as ZT12. For animals housed in DD, the daily onset of wheel running was used to estimate circadian time 12 (CT12).

### Tissue collection

Animals housed in running wheel cages were sacrificed at six defined times of day under either LD following stable entrainment or DD after stable free-running behaviour was established: ZT and CT 1, 5, 9, 13, 17 and 21, respectively (n = 12 per time of day). For *Bmal1^−/−^* mice, animals were transferred into DD at ZT12 and sacrificed at 17 h and 29 h later, reflecting ZT5 and ZT17 in the following circadian cycle. True circadian time cannot be established in these animals because behavior reflects their inherent molecular arrhythmicity in the absence of a daily light cue. Since photic stimulation of the SCN may itself stimulate expression of Runx2, animals were sampled within the first day without photic stimulation in order to sample tissues prior to the breakdown of entrained rhythms.

For tissue collection for gene or protein expression analyses, animals were overdosed with sodium pentobarbital; brains were rapidly removed and deeply frozen under sterile RNase-free conditions. Microdissections of distinct brain regions were performed by isolating tissue from frozen coronal sections using a sterilized blunt-end needle. Brain regions included olfactory bulb, suprachiasmatic nucleus (SCN), paraventricular nucleus of the hypothalamus (PVN) and hippocampus (HP). Tissue punches were stored at −80°C until further processed.

For immunohistochemistry analyses, animals were deeply anaesthetized with sodium pentobarbital and perfused transcardially with 50 mL of 4% paraformaldehyde in 0.1 M phosphate buffer. Brains were removed, post-fixed in 4% paraformaldehyde for 4 h at 4°C and then stored in 20% sucrose in 0.1 M sodium phosphate buffer (PB) with 0.01% sodium azide. Brains were cut using a freezing microtome into 4 parallel series of 25 µm thick coronal sections and stored at −20°C in cryoprotectant [23].

### Total mRNA isolation and real-time polymerase chain reactions

RNA samples were produced by pooling tissue isolated from three mice in the same treatment group to generate a total of n = 4 per time point. Tissue samples were homogenized and total RNA isolated using TriZol reagent (Invitrogen, Burlington, ON). Reverse transcription of total RNA was performed using the High Capacity cDNA Reverse Transcription Kit (Applied Biosystems Inc., Foster City, CA) according to the manufacturer's instructions. Confirmation of gene expression in the SCN was performed by qualitative RT-PCR conducted in triplicate using 1 µg cDNA, 15 µL of QuantiFast Probe PCR Kit Master Mix (Qiagen, Mississauga, ON) and 3 µL of each forward and reverse primer for a total PCR reaction volume of 30 µL. Sequences of forward and reverse primers: *Runx2* forward: 5′-CCGCACGACAACCGCACCAT-3′ and reverse: 5′-CGCTGGGGCCCACAAATCTC-3′ amplifies 289 bp product; *Runx1* forward: 5′-CATCCGGTCTCCACTCAGTT-3′ and reverse: 5′-TGGCTTATGGGTCTTCCTTG-3′amplifies 612 bp product; *Runx3* forward: 5′-CAGGTTCAACGACCTTCGAT -3′ and reverse: 5′-GAGGTAGGTGTGGTGGAAGC-3′ amplifies 517 bp product; *Osteocalcin* forward: 5′-CTCACCCTGTCCCCTAAGC-3′ and reverse: 5′-CCGTAGATGCGTTTGTAGGC-3′ amplifies 489 bp product (OligoCore, The University of Western Ontario, London, ON, Canada). Reactions were preceded by an initial denaturation step (5 min) followed by 35 amplification cycles of 45 s denaturation at 95°C, 45 s annealing at 60°C and 1 min elongation at 72°C with a final 10 min elongation. PCR products were separated by electrophoresis on a 1% agarose gel in 1× TAE buffer. Gels were visualized and images captured using the MultiImage Light Cabinet (Alpha Innotech Corporation, Santa Clara, CA). The genomic identity of amplified sequences having predicted lengths were confirmed by molecular sequencing (Robarts Research Institute, the University of Western Ontario, Canada).

For quantitative analysis of gene expression, real-time qRT-PCR reactions were performed using 2 µL of cDNA, 10 µL of TaqMan Universal PCR Master Mix 2× (Applied Biosystems), 0.8 µL of 25 mM MgCl_2_ (Invitrogen), 6.2 µL DEPC and 1 µL of TaqMan gene expression assay solution for the gene of interest. QRT-PCR reactions were performed for Bmal1 (Mm00500226_m1), Per1 (Mm00501813_m1), Per2 (Mm00478133_m1), Runx2 (Mm00501578_m1), Runx1 (Mm01213404), Osteocalcin (OC; Rn01455285_g1), Osteopontin (Mm01611440_mH) and Glyceraldehyde-3-phosphate dehydrogenase (GAPDH; Rn99999916_s1). Triplicate reactions for each sample were performed using a Rotor-Gene RG-6000 thermocycler and software (Corbett Life Sciences, San Francisco, CA). Each reaction was performed using the following PCR conditions: hold at 50°C for 2 min and 95°C for 10 min, 40 cycles of 95°C for 15 sec and 58°C for 1 min. The cycle threshold (C_t_) values were determined from an optimal threshold line generated from standard curve dilutions for each gene analyzed. In brief, a series of 5-log dilutions of cDNA reverse transcribed from RNA isolated from the brain region of interest was used to perform qPCR reactions for each probe to be used for analyses. Standard curves were deemed optimal when the line of best fit through the C_t_ values of the dilutions had a R^2^≥0.99 and an efficiency value between 0.8–1. The C_t_ of each experiment sample was determined from the intersection between the baseline subtracted amplification curve of the sample and the threshold line established by the optimal standard curve. The gene of interest levels of expression within a sample was normalized to their respective levels of the expression of the housekeeping gene, glyceraldehyde-3-phosphate dehydrogenase (GAPDH), within each sample; this value corresponds to the ΔC_t_ value. In the current study, the ΔΔC_t_ value represents the difference between gene expression in an individual sample at a given time of day and the overall average level of gene expression measured at all-time points sampled rather than a comparison to a control group as typically performed (ΔC_t(average)_−ΔC_t(sample) = _ΔΔC_t_). Relative mean fold changes for each sample were calculated relative to the average ΔΔC_t_ across samples at all times of day sampled with the value 1 representing a baseline level of expression. One-way Anovas and two-tailed t-tests were conducted to identify significant daily rhythms of each gene within each brain region, with significance levels of *p*<0.05.

For analyses of SCN tissues from *Bmal1* mutant mice, data were normalized to GAPDH levels and are represented as a relative mean fold change from the average gene expression detected at both time points with the value 1 representing a baseline level of expression. One-way Anovas and two-tailed t-tests were conducted to identify significant daily rhythms. Values were considered significantly different with *p*<0.05.

### Total protein isolation and western blotting

Protein samples were generated by pooling tissue isolated from three mice in the same treatment group to generate a total of n = 4/time point (CT9 and CT21). Methodology applied for protein isolation, preparation of protein lysates, PAGE, electrophoretic blotting and immunodetection of proteins of interest has been previously described [24]. In brief, protein samples of equal volume and concentration were loaded on a 10% polyacrylamide gel and separated under reducing conditions [24]. All samples were run in duplicate and balanced by time point and genotype across individual gels. Precision Plus Protein All Blue Standards (Bio-Rad Laboratories Ltd.) were used as molecular weight markers. Following protein transfer onto Millipore Immobilon-FL polyvinylidene difluoride membranes (Millipore, Billerica, MA), membranes were individually incubated for 16 h on a shaker at 4°C with an antibody raised against the loading control protein, GAPDH (mouse anti-GAPDH; 1∶20,000; MAB374, Chemicon International, Temecula, CA), and one of the following rabbit anti-mRunx2 (mouse) antibody M-70 (1∶200; Santa Cruz Biotechnology Inc., Santa Cruz, CA); and rabbit anti-Bmal1 (1∶200; PA1-523, Affinity BioReagents, Golden, CO. The following secondary antibodies were used to visualize immunolabeled blots: IRDye 800CW-conjugated polyclonal goat anti-mouse (1∶20,000; LI-COR Biosciences, Lincoln, NE); Alexa 680-conjugated polyclonal goat anti-rabbit (1∶5000; Invitrogen); and IRDye 800CW-conjugated polyclonal donkey anti-mouse (1∶20,000; LI-COR Biosciences). Images were captured using an Odyssey 2.1 scanner (LI-COR Biosciences). Quantification of protein concentration was performed by determining ratios of protein of interest/GAPDH intensity levels for each lane and averages of the duplicates were calculated for each protein sample. The overall fold change in protein across the day was determined by normalizing the ratios for each sample to the overall mean at CT9 of the control (WT) groups. Post hoc Tukey analyses were performed where appropriate to determine time of day effects and values were considered significantly different with *p*<0.05.

**Figure 1:**
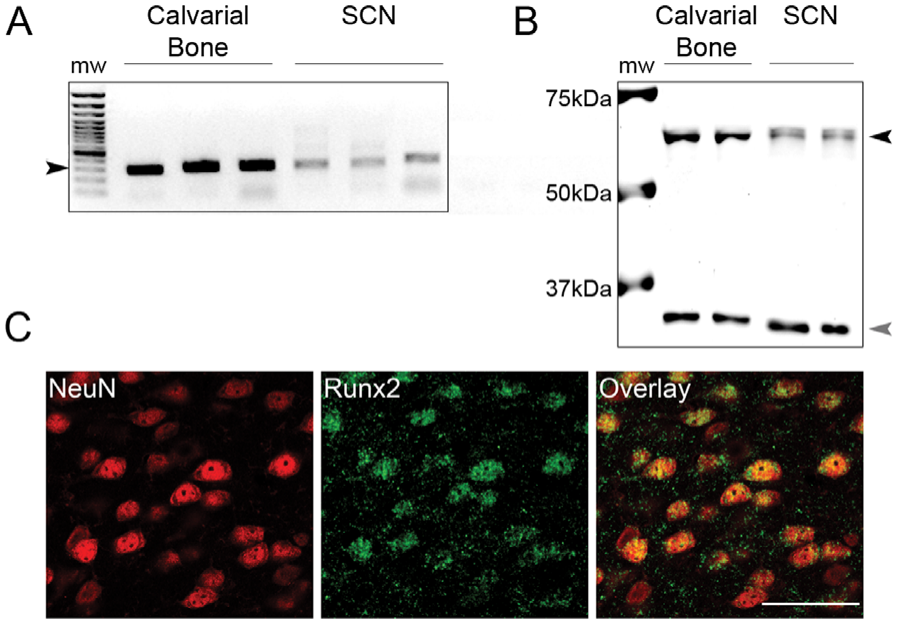
Runx2 gene and protein expression is detectable in neurons of the adult mouse brain. A) Qualitative PCR analysis of Runx2 mRNA (289 bp) expression in the suprachiasmatic nucleus (SCN) of adult mice. Lane 1: 100 bp molecular weight standard ladder (black arrowhead: 300 bp; grey arrowhead: 500 bp). Lane 2–4: Runx2 (289 bp) PCR product from reactions performed using E15 mouse calvarial bone mRNA extracts and Runx2-specific primers. Lane 5–7: Runx2 (289 bp) PCR product from reactions performed using adult mouse SCN mRNA extracts and Runx2-specific primers. Sequencing confirmed the identity of all positive PCR amplification products. B) Western blot analysis of Runx2 protein (∼60 kDa; black arrowhead) expression in the SCN of adult mice. Lane 1: Molecular weight standard ladder. Lane 2–3: protein isolated from E15 mouse calvarial bone. Lane 4–5: protein isolated from SCN of adult mice. GAPDH (∼37 kDa; grey arrowhead) protein detection served as a loading control. C) Immunolabelling of Runx2 (green) and NeuN (red) in the frontal cortex. Overlay image showing co-localization of Runx2 and NeuN (yellow). Scale bar: 50 µm.

All primary antibodies resulted in single bands of immunoreactivity at their respective expected molecular weights. Labeling of western blots of protein isolated from calvarial bone and SCN tissue with the Runx2 antibody resulted in labeling of a single band at the predicted molecular weight for Runx2 (∼60 kDa; Figure 1B) further confirming the specificity of the antibody recognition over Runx1 (∼50 kDa) and Runx3 (∼45 kDa).The specificity of the Runx2 antibody used in the current study has been previously documented [25]–[27].

### Immunohistochemistry

For immunohistochemical analyses, a series of sections throughout the forebrain, including sections containing the SCN and PVN, from perfused tissue was processed for Runx2. Immunostaining was performed at room temperature, with gentle agitation of the free-floating sections, and with extensive rinses with 0.1 M phosphate buffered saline, (pH 7.35; PBS) between incubation steps. First, sections were incubated for 10 min in 1% H_2_O_2_ and for 1 h in incubation solution (PBS containing 0.4% Triton-X100 (PBSTX; Fisher Scientific, Inc.; Ottawa, Canada) and 0.1% bovine serum albumin fraction V (BSA; Fisher Scientific)). Sections were then incubated in rabbit antibody raised against mouse Runx2 (1∶200 in incubation solution; M-70, Santa Cruz Biotechnology) for 16 h, in biotinylated goat anti-rabbit secondary antibody (1∶500 in PBSTX+BSA; Vector, Burlington, ON) for 1 h and in ABC-elite (1∶1000 in PBS; Vector) for 1 h. Reaction product was visualized with 3,3′-diaminobenzidine (0.2 mg/ml in 0.1 M PB; Sigma Oakville, ON) dissolved in PB supplemented with 0.0004% H_2_O_2_ for 10 min. Sections were mounted onto positively charged glass slides (Fisher Scientific), dehydrated in an increasing ethanol gradient, delipidated with CitriSolv (Fisher Scientific) and cover-slipped with DPX mounting medium (Electron Microscopy Sciences, Hatfield, PA).

For immunofluorescence, sections were incubated in rabbit antibody raised against mouse Runx2 (1∶200 in PBSTX+BSA) for 16 h, in biotinylated goat anti-rabbit (1∶500 in PBSTX+BSA; Vector, Burlington, ON) for 1 h, in ABC-elite (1∶1000 in PBS; Vector) for 1 h, in biotinylated tyramine (PerkinElmer, Waltham, MA; 1∶250 diluted in PBS with 3% H_2_O_2_) for 10 min, and in Alexa 488-conjugated streptavidin (1∶200 in PBS; Molecular Probes) for 30 min. Sections were next incubated in mouse anti-NeuN (1∶1000 in PBSTX+BSA; MAB377, Chemicon International) for 16 h and Alexa 555-conjugated goat anti-mouse (1∶100 in PBSTX+BSA; A-21424, Molecular Probes) for 30 min. Sections were mounted onto positively charged glass slides, dried and cover-slipped with an aqueous mounting medium (Gelvatol) containing anti-fading agent 1,4-diazabicyclo(2,2)octane (DABCO; 50 mg/ml, Sigma-Aldrich, St. Louis, MO). Immunohistochemical controls included the omission of individual primary antibodies resulting in absence of specific immunolabeling for the corresponding antigen.

### Histological analysis

To determine the global distribution of Runx2, a qualitative analysis was performed of Runx2 immunoperoxidase labeled sections of wild type mice (n = 4 at CT5 and CT9 each). Optical density analyses of Runx2 immunoperoxidase labeled sections were conducted using images of the SCN and PVN that were captured with a Leica DFC420 camera connected to a Leica DM5000B microscope (Leica Microsystems, Wetzlar, Germany). All images (2–3 images per animal/area of interest) were captured using identical microscope and camera settings. Density analyses of immunolabeled cells were conducted using ImageJ software (NIH, Bethesda, MD) with fixed threshold settings and were expressed as the area above threshold. Mean areas above threshold (± SEM) were calculated for each area in individual animals and used to calculate the mean over all animals (± SEM) for each brain region at a given time of day (CT1, 5, 9, 13, 17, and 21). The data were expressed as the mean fold change relative to the overall mean across times of day analyzed (± SEM; 6 time points). Anova analyses were performed to determine the overall effects of time of day followed by Tukey post hoc tests for pair-wise comparisons between each time of day when appropriate. Values were considered significantly different when *p*<0.05.

### Confocal Microscopy

Cortical tissues were imaged and captured from coronal slice of adult mouse brain (25 µm) using a Zeiss LSM-510 ConfoCor laser scanning confocal microscope system (Carl Zeiss MicroImaging GmbH, Göttingen, Germany). Alexa 488 fluorescence (green) was imaged with a 505–530 nm band pass filter and Argon laser and Alexa 555 fluorescence (red) with a 560–615 nm band pass filter and a HeNe1 laser. Z-stack images of 1.0 µm thick optical sections through sampled sections were captured with x63 oil immersion objective. Images were used for qualitative analyses only. Colocalization of NeuN and Runx2 immunostaining in cells was determined based on overlapping fluorescence detected in overlays of images captured from the same Z-stack images with each fluorescence filter. Images were viewed using Zeiss LSM Image Browser (Carl Zeiss MicroImaging GmbH) and were not altered in any way except for adjustment of brightness.

### *In vitro* monitoring of mPer2^Luc^ bioluminescence

For bioluminescence studies, *mPer2^Luc+/+^*/*Runx2^+/−^* were mated and pups collected on embryonic day 19–20 (E19–20). Runx2 expression has previously been described in neonatal brain tissue [28]. Dames were deeply anesthetised with CO_2_, decapitated and uterine sacs containing pups were removed and placed in 4°C HBSS (Sigma). Pups were individually dissected out of the uterine sac, brain removed and encased in 4% low-melt agar (Fisher Scientific). Hypothalamic coronal slices (300 µm) containing the SCN were cut on a vibrotome (ThermoScientific, Burlington, ON) at 4°C and the SCN was isolated with the aid of a microscope. Tissues were placed on individual Millicell organotypic culture plate inserts (Millipore, Etobicoke, ON) in a 35 mm Petri dish (BD Biosciences, Mississauga, ON) with 1.2 ml of culture media [DMEM (Sigma) supplemented with 0.35 g/L sodium bicarbonate (Sigma), 10 mM HEPES (pH 7.2, Sigma) B27 (2%; Invitrogen), 0.1 mM luciferin (Promega, Madison, WI), and antibiotics (1000 U/ml penicillin, 1000 µg/ml streptomycin; Gibco, Burlington, ON)] For additional methodological details, see [29]. Livers from each animal were dissected out and sections ∼1–2 mm^3^ were cut by hand and cultured separately. Cultures were maintained at 36.5°C and bioluminescence was measured using a LumiCycle (Actimetrics, Evanston, IL).

Bioluminescence data analyses were performed using the LumiCycle Analysis Program (Actimetrics, Wilmette, IL). Cycle 0 data (slice preparation start through midnight of the first day) were excluded from the period analyses and only recordings containing at least three mPer2^Luc^ peaks were used for analyses. Significant rhythms were determined by χ^2^ periodogram analyses conducted following subtraction of a baseline determined by fitting a low-order polynomial to each data set. Statistical analyses of period length and amplitude of bioluminescence were performed using the baseline-subtracted rhythms. Differences between genotypes were determined using one-way Anovas and Tukey post hoc analyses were conducted where appropriate. Values were considered significantly different with *p*<0.05. Genotype of pups was determined using gDNA extracts from tail biopsies performed during brain dissection (see *Animals* section for genotyping details).

### Behavioral analysis of *Runx2^+/−^* mice

Behavioral entrainment of *Runx2* ^+/−^ mice to a LD schedule and ability to establish stable free-running activity rhythms in constant darkness (DD) were assessed and compared to age and sex-matched *Runx2^+/+^* littermates. Behavioral analyses of *Runx2^−/−^* mice could not be evaluated due to the fact that these animals lack proper ossification and do not survive past post-natal day 1 [21]. For phase shifting studies, animals were subjected to an Aschoff type II phase shifting paradigm (Mrosovsky, 1996). In brief, animals were initially housed under a strict 12 h∶12 h LD schedule for 7 days, subsequently released into constant dim red illumination at ZT12 (lux<5), and left undisturbed (control) or given a 15 min light pulse (same intensity as for light period during 12 h∶12 h LD entrainment) at CT6, CT15, or CT21. Following each manipulation, the animals remained in DD for 7 days. All animals were exposed to each treatment and were allowed to stably re-entrain for at least 7 days prior to the each photic pulse.

Phase shift responses to light were calculated from running wheel activity records. CLOCKLAB software (Actimetrics) was used to detect the daily onset of wheel running and to fit a regression line to the 7 daily onsets prior to the photic manipulation. Phase shift magnitude (hours ± SEM) was calculated by subtracting the observed activity onset on day 6 (phase advance) or day 3 (phase delay) of DD from the predicted onset prior to light treatment. Positive values represent phase advances, negative values represent phase delays. To control for individual variability in free-running period, the magnitude of the phase shift produced by the pulse treatments was subtracted by the inherent shift observed in each animal following the no light control condition. Mean normalized phase shift values for each treatment and genotype group were determined and comparisons between genotypes for a given photic manipulation were conducted using one-way Anova and paired t-test analyses.

## Results

### Expression of *Runx2* in the mouse brain

Given the limited description of the expression of *Runx2* in the adult mammalian brain [19], we first established whether detectable levels of *Runx2* are expressed in the adult C57BL/6 mouse SCN. Qualitative RT-PCR assays validated expression of *Runx2* in this brain region (Figure 1A). Consistent with previous observations that expression of *Runx1* is ubiquitously expressed in the mammalian central nervous system in contrast to *Runx3*, another member of this transcription factor family, RT-PCR assays detected expression of *Runx1* in SCN samples; however, *Runx3* was undetectable. In addition, *Osteocalcin*, a known target of *Runx2* transcription factor activity [30], was detected in the SCN (not shown). However, a second well established target of Runx2 in osteoblasts, *Osteopontin* [31], was not found to be expressed in the SCN. These findings suggest that the *Runx2* gene product might have distinct functionality in the murine SCN compared to bone.

**Figure 2:**
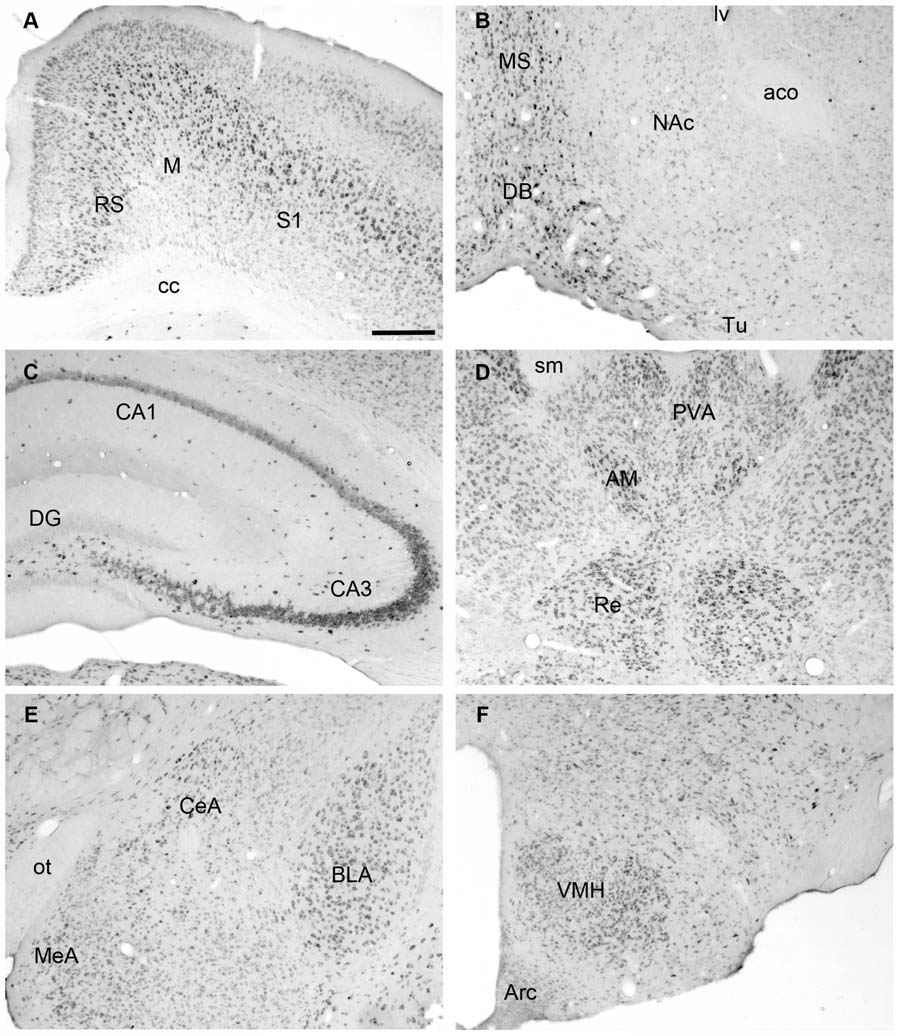
Examples of RUNX2 immunolabeling in the adult mouse brain. RUNX2 immunlabelling in the cortex (A), ventral striatum (B), hippocampus (C), thalamus (D), amygdala (E), and hypothalamus (F) in a representative adult male mouse at CT9. Scale bar indicates 250 µm. Abbreviations: aco: anterior commissure; AM: anteromedial nucleus of the thalamus; ARC: arcuate nucleus; BLA: basolateral nucleus of amygdala; cc: corpus callosum; CeA: central nucleus of amygdala; DB: diagonal band of Broca; DG: dentate gyrus; lv: lateral ventricle; M: motor cortex; MeA: medial nucleus of amygdala; MS: medial septum, NAc: nucleus accumbens; ot: optic tract; PVA: paraventricular thalamic nucleus, anterior; Re: reuniens thalamic nucleus; RS: retrosplenial cortex, S1: somatosensory cortex; sm: stria medularis; Tu: olfactory tubercle; VMH: ventromedial nucleus of hypothalamus.

**Figure 3:**
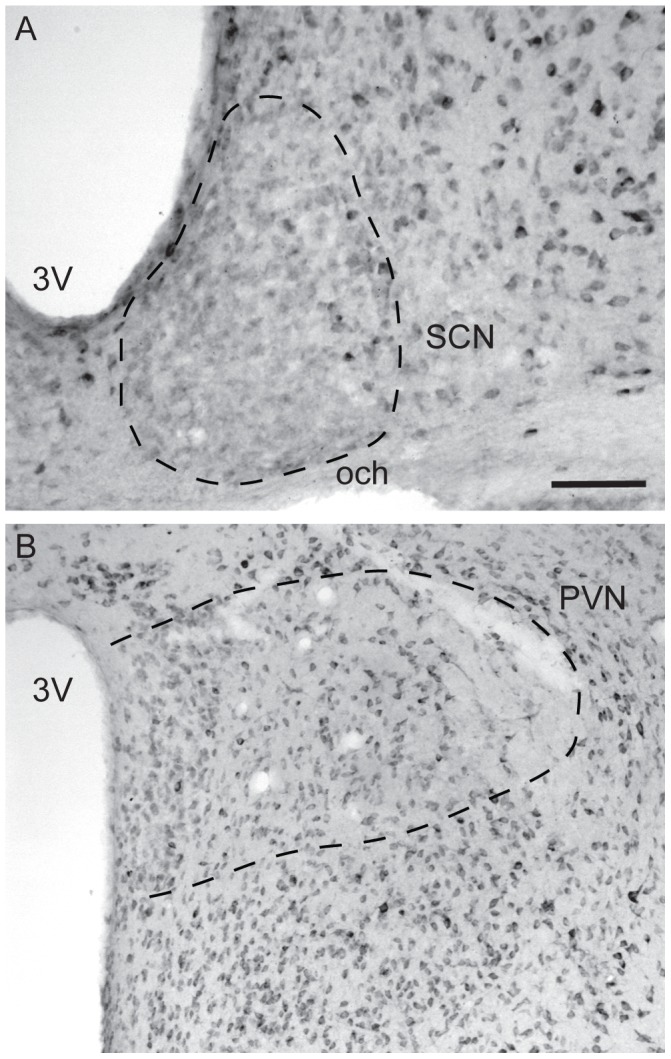
Example of RUNX2 immunolabeling in the suprachiasmatic nucleus (SCN) and paraventricular nucleus (PVN) in a representative adult male mouse at CT9. The dotted lines outline the area of interest, the SCN in A and the PVN in B. Scale bar depicts 125 µm in A and 250 µm in B. Abbreviations: 3V: third ventricle; och: optic chiasm; PVN: paraventricular nucleus; SCN: suprachiasmatic nucleus.

Next, we determined whether RUNX2 protein was detectable in the SCN of C57BL/6 mice. Western blot analyses of protein extracts from the SCN of adult mice resulted in a band of immunoreactivity at the predicted molecular weight of RUNX2 (∼60 kDa) (Figure 1B) with a corresponding band also present in tissue isolated from mouse calvaria on embryonic day 15 mice [32]–[34]. In order to determine if RUNX2 is expressed by neurons, coronal sections of fixed mouse brain tissue were immunolabeled for RUNX2 protein and the neuronal nuclei marker, NeuN [35]. Confocal analysis revealed that RUNX2-positive staining was exclusively localized to NeuN-positive cells in all brain areas (Figure 1C). As others have observed *Runx2* promoter activity in several brain structures including the cortex and hippocampus [19], we also determined whether RUNX2 protein was detectable in these and other brain areas. A qualitative analysis of RUNX2 distribution in the brain revealed that RUNX2 is not homogenously expressed. Specifically, dense immunolabeling was observed in all areas of the cortex (Figure 2A), except piriform cortex, where labeling was light to moderate. Dense labeling was also observed throughout the thalamus (Figure 2D) and in numerous areas within the hypothalamus, including the paraventricular nucleus of the hypothalamus (PVN), SCN, ventromedial hypothalamus (Figure 2F), preoptic area, anterior and later hypothalamus, but was absent in the arcuate nucleus and light in dorsomedial nucleus of the hypothalamus. Closer analysis of immunolabeling in the SCN and PVN revealed homogenous distribution of RUNX2 in both of these areas important in regulation of circadian functions (Figure 3). Moderate labeling was evident in the amygdala (Figure 2E), but was light or absent in the bed nucleus of the stria terminalis and lateral septum. RUNX2 immunolabeling was absent or light throughout the dorsal and ventral striatum (Figure 2B), including caudate putamen, nucleus accumbens, and globus pallidus. RUNX2 was present in the hippocampus, with dense labeling in CA3, and light scattered labeling in CA1–2 and dentate gyrus (Figure 2C). Finally, immunolabeling for RUNX2 was dense in most areas of the midbrain and brainstem, including ventral tegmental area and substantia nigra, dorsal raphe and red nucleus.

### *Runx2* gene expression is rhythmic in the mouse brain

**Figure 4:**
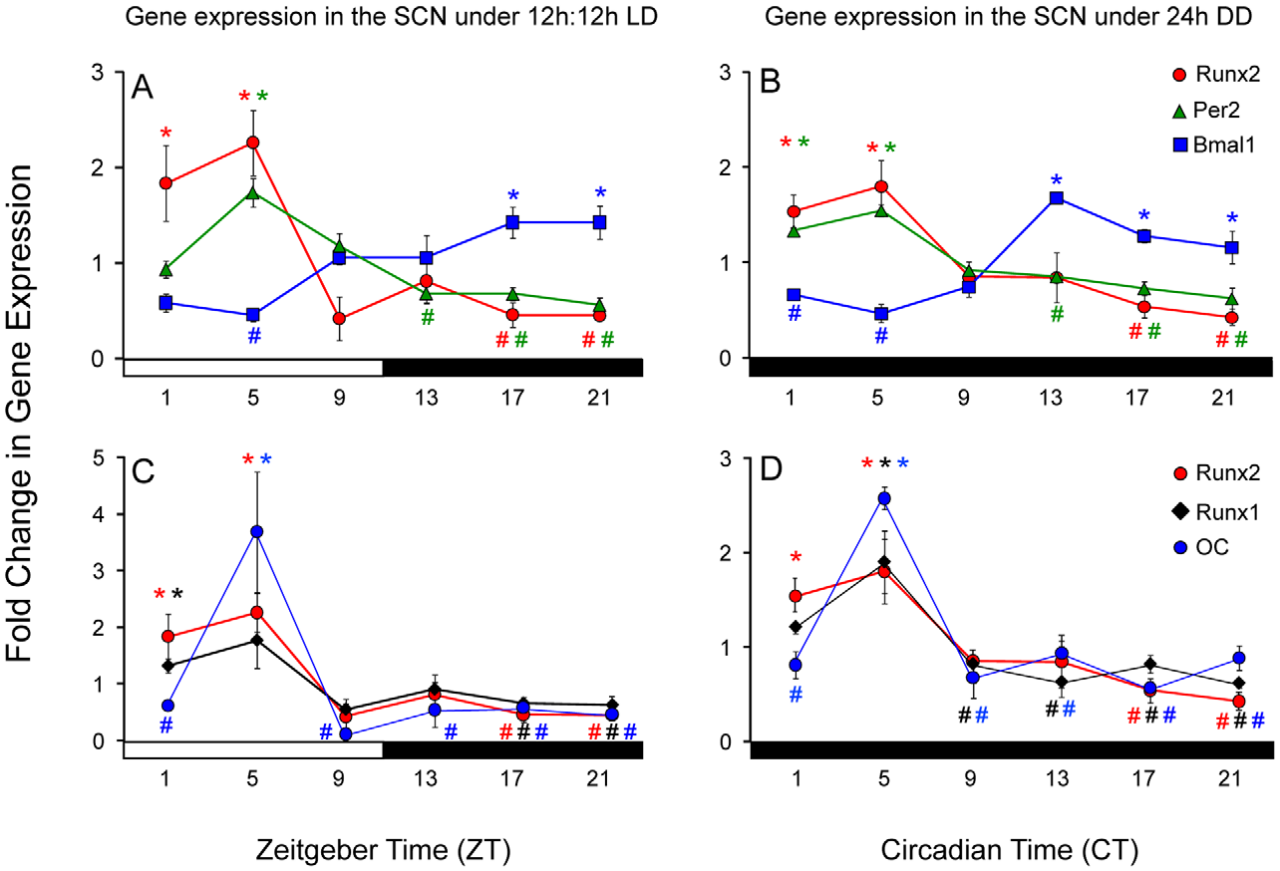
Runx2 gene expression is rhythmic in the SCN of wild type mice. Fold changes in levels of gene expression (± SEM) in the SCN for Runx2 (red circle), Per2 (green triangle; A and B), Bmal1 (blue square; A and B) and Runx1 (black diamond; C and D) and OC (blue circle; C and D) determined by real-time qRT-PCR under 12 h∶ 12 h LD (A and B) and 24 hr DD (C and D). Values represent the level of normalized gene expression relative to the mean overall normalized gene expression across the day. In 12∶12 h LD conditions, time of day is represented by white and black horizontal bars indicating periods of day (light) and dark (night), respectively, with dark onset marking ZT12. In 24 h DD conditions, black horizontal bars represent darkness during subjective day and night with running wheel activity onset marking CT12. Runx2 gene expression data for 12∶12 h LD and 24 h DD were plotted in both panels to allow for comparison with multiple gene profiles. Significant peaks levels of expression (*) compared to nadir points (#) for given gene presented in respective color (*p*<0.05).

To examine the circadian regulation of *Runx2*, gene expression was examined in the SCN and several other brain regions previously shown to be rhythmic under both LD and DD conditions [36]. In the SCN, under LD conditions, there was a significant main effect of time of day upon *Runx2* expression (F (5, 20) = 5.652; *p*<0.01) (Figure 4). Post hoc analyses revealed significantly greater expression of *Runx2* during the early to mid-day than during the night (ZT1 vs. ZT17 and 21, *p* = 0.03 and 0.009; ZT5 vs. ZT13, 17 and 21, *p* = 0.025, 0.009, 0.0017; Figure 4A). The rhythmic expression of *Per2* (F (5, 21) = 6.439; *p*<0.005) was in phase with the *Runx2* rhythm, with peak levels observed during midday (ZT5 vs. ZT13 and 21, *p* = 0.004 and 0.0014; Figure 4A). Rhythmic expression of *Bmal1* (F (5, 21) = 14.171; *p* = 0.015) was in antiphase compared to the expression of both *Per2* and *Runx2*, with greater levels of *Bmal1* expression occurring during the night than day (ZT5 vs. ZT17–21, *p* = 0.002; ZT5 vs. ZT13, *p* = 0.05; Figure 4A).

Under DD conditions, *Runx2* expression in the SCN was also rhythmic (F (5, 21) = 6.117; *p* = <0.005) with peak levels in the early to midday (CT1–5) and a nadir in the mid to late night (CT1 vs. CT17 and 21, *p* = 0.004 and 0.0016; CT5 vs. CT17 and 21, *p* = 0.004 and 0.003; Figure 4B). Likewise, the *Per2* expression rhythm (F (5, 20) = 16.509; *p* = <0.001) was similar in phase to that observed for *Runx2* (peak levels observed during early to midday; CT1 vs. CT13, 17 and 21, *p* = 0.0007, 0.0011 and 0.0033; CT5 vs. CT13, 17 and 21, *p* = 0.0003, 0.0001 and 0.0003) and antiphase to the rhythm of *Bmal1* (peak in late night; CT1 vs. CT13, 17 and 21, *p* = 0.000004, 0.0008 and 0.009; CT5 vs. CT13, 17 and 21, *p* = 0.00005, 0.0005 and 0.0123; CT9 vs. CT13 and 17, *p* = 0.0002 and 0.007; Figure 4B). In addition, another member of the period gene family, *Per1*, was also expressed in phase with *Runx2*, with peak levels observed during early to midday (CT1 vs. CT13 and 21, *p* = 0.0046 and 0.0046; CT5 vs. CT13, *p* = 0.003).

To address whether other genes associated with Runx2 are also rhythmic, gene expression of *Runx1* and *Osteocalcin* was examined under LD and DD. Rhythmic expression of *Runx1* (F (5, 20) = 6.209; *p* = <0.005) and *Osteocalcin* (F (5, 20) = 11.881; *p* = 0.036) was evident under 12 h∶12 h LD conditions with peaks in early to midday (*Runx1*: ZT1 vs. ZT17 and 21, *p* = 0.006 and 0.011; *Osteocalcin*: ZT5 vs. ZT1, 5, 9, 13, 17 and 21, *p* = 0.004, 0.003, 0.0016 and 0.001; Figure 4C). Rhythmic gene expression was also observed in DD for *Runx1* (F (5, 22) = 12.883; *p* = 0.008) and *Osteocalcin* (F (5, 21) = 7.113; *p* = 0.001) with significant peaks in expression in midday, approximately 4 h following the observed initial rise in *Runx2* expression observed under DD conditions (*Runx1*: CT5 vs.CT9, 17 and 21, *p* = 0.012, 0.013, 0.016 and 0.006; *Osteocalcin*: CT5 vs.CT9, 13, 17 and 21, *p* = 0.027, 0.029, 0.011 and 0.043; Figure 4D).

**Figure 5:**
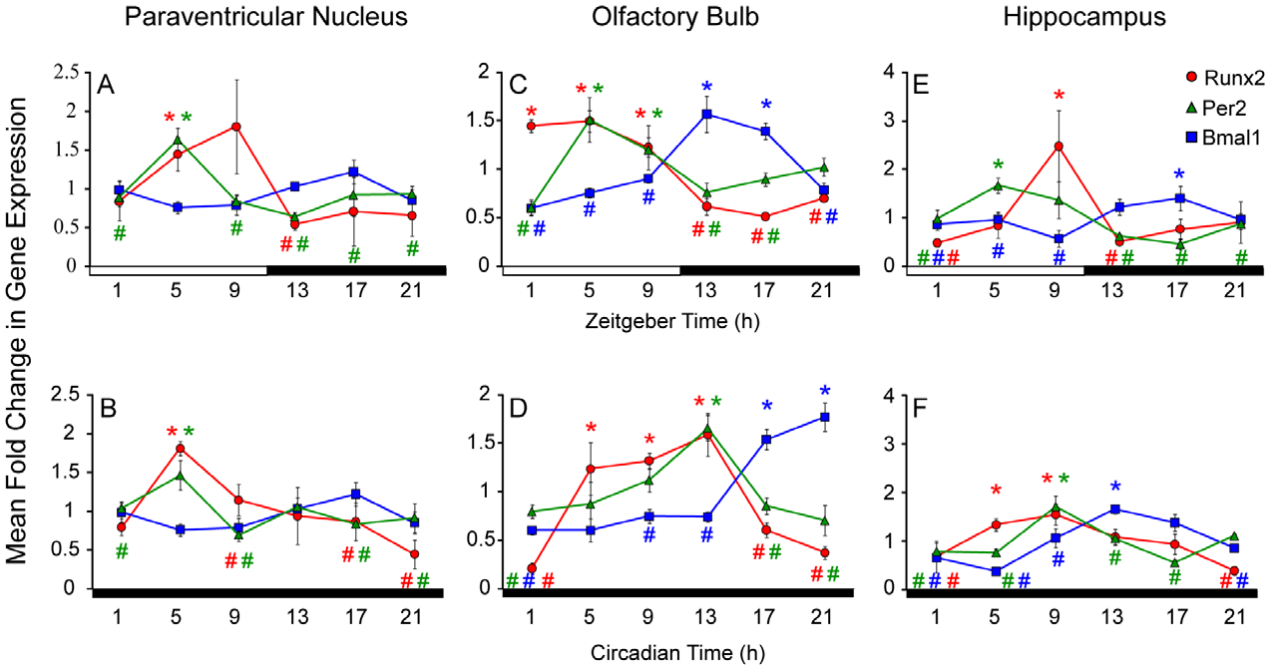
Runx2 gene expression is rhythmic in the brain regions outside of the SCN. The paraventricular nucleus, the olfactory bulb and the hippocampus tissues were assayed fewer than 12∶12 h LD (A, C and E) and 24 h DD (B, D and F) conditions. Fold changes in levels of gene expression (± SEM) for Runx2 (red circle), Per2 (green triangle) and Bmal1 (blue square) determined by real-time qRT-PCR. In 12 h∶12 h LD conditions, time of day is represented by white and black horizontal bars indicating periods of day and night, respectively, with dark onset marking ZT12. In 24 h DD conditions, grey and black horizontal bars represent subjective day and night, respectively, with running wheel activity onset marking CT12. Significant peaks levels of expression (*) compared to nadir points (#) for given gene presented in respective color (*p*<0.05).

Expression of *Runx2* and the clock genes *Per2* and *Bmal1* were also examined in the paraventricular nucleus (PVN), olfactory bulb (OB) and hippocampus (HP) under LD and DD conditions. Rhythmic *Runx2* gene expression was observed under both lighting conditions in the PVN (LD: F (5, 21) = 7.364, *p* = 0.002; DD: F (5, 21) = 3.914, *p* = 0.022), OB (LD: F (5, 20) = 1.535, *p* = <0.001; DD: F (5, 22) = 16.455, *p* = <0.001) and HP (LD: F (5, 22) = 74.407, *p* = 0.009; DD: F (5, 20) = 14.074, *p* = 0.008; Figure 5). In the PVN, expression of *Per2* was rhythmic under LD (F (5, 19) = 4.683, *p* = 0.01) and DD conditions (F (5, 19) = 3.940, *p* = 0.021; Figure 5A and B). *Per2* expression mirrored that of *Runx2* with greatest levels detected during mid to late day and nadirs observed during the night under both conditions (Figure 5A and B). The peaks in *Runx2* and *Per2* expression in the PVN were phase delayed by 4 h compared to the SCN (Figure 4A and B, 5A and B). In contrast to the SCN, *Bmal1* expression was not rhythmic in the PVN under LD conditions (F (5, 20) = 1.856, *p* = 0.162) with a trend for rhythmicity in DD (F (5, 19) = 3.041, *p* = 0.058; Figure 5A and B). As in the SCN, *Bmal1* appeared to be in antiphase to *Per2* and *Runx2* in the PVN under DD conditions (Figure 5B).

The phase relationship between *Runx2* and *Per2* expression observed in the OB under LD conditions was similar to that seen in the SCN and PVN but was not as tightly linked when examined under DD. Specifically, the rhythmic expression of *Per2* rhythm in the OB under LD conditions (F (5, 22) = 8.455, *p* = <0.001) was similar to that of *Runx2* (Figure 5C). However, the *Per2* expression peak was phase delayed by 4 h compared to the SCN while the *Runx2* peak did not shift (Figure 5C). In DD, *Runx2* expression was shifted by 4 h compared to detected levels in the SCN and elevated levels were extended over the mid day to the early night in the OB in DD (Figure 5D). In contrast, the peak in *Per2* rhythm (F (5, 21) = 7.406, *p* = <0.001) was delayed by 8 h under DD compared to its peak expression detected under LD conditions (Figure 5C and D) and 12 h compared to its peak in the SCN (Figure 4A and B). Rhythmic expression of *Bmal1* (LD: F (5, 21) = 18.066, *p* = <0.001; DD: F (5, 23) = 28.053, *p* = <0.001) also displayed differing shifts in expression under LD and DD conditions in the OB and maintained an antiphase relationship with *Runx2* and *Per2* under both conditions.

In the HP, *Per2* was rhythmic under LD (F (5, 23) = 15.902, *p* = 0.01) and DD conditions (F (5, 19) = 8.206, *p* = <0.001; Figure 5E and F). As observed in the PVN, the peak in *Per2* expression was shifted by 4 h in the HP compared to the SCN. This peak was 4 h earlier than the peak in *Runx2* expression, demonstrating a difference in phase between *Per2* and *Runx2* in the HP under LD conditions (Figure 5E). *Bmal1* expression appeared to be in antiphase to *Per2* under LD conditions but displayed only a trend toward rhythmicity in this brain area (F (5, 20) = 2.675, *p* = 0.06; Figure 5E). *Per2* expression was also rhythmic in the HP under DD conditions (F (5, 20) = 8.206, *p* = <0.001) and displayed a similar phase in expression to *Runx2*, with shifts in peak levels of expression by 4–8 h compared to the SCN. Expression levels of *Runx2* remained elevated for a longer interval than *Per2* levels (Figure 5F). This was opposite of what was observed under LD conditions wherein *Per2* had prolonged elevated levels of detectible expression compared to a single clear peak for *Runx2* (Figure 5E). Under DD conditions, *Bmal1* expression was rhythmic (F (5, 20) = 13.226, *p* = <0.001) with peak levels in late night and was antiphase to *Runx2* and *Per2* rhythms (Figure 5F).

### *Runx2* protein expression is rhythmic in the mouse SCN

**Figure 6:**
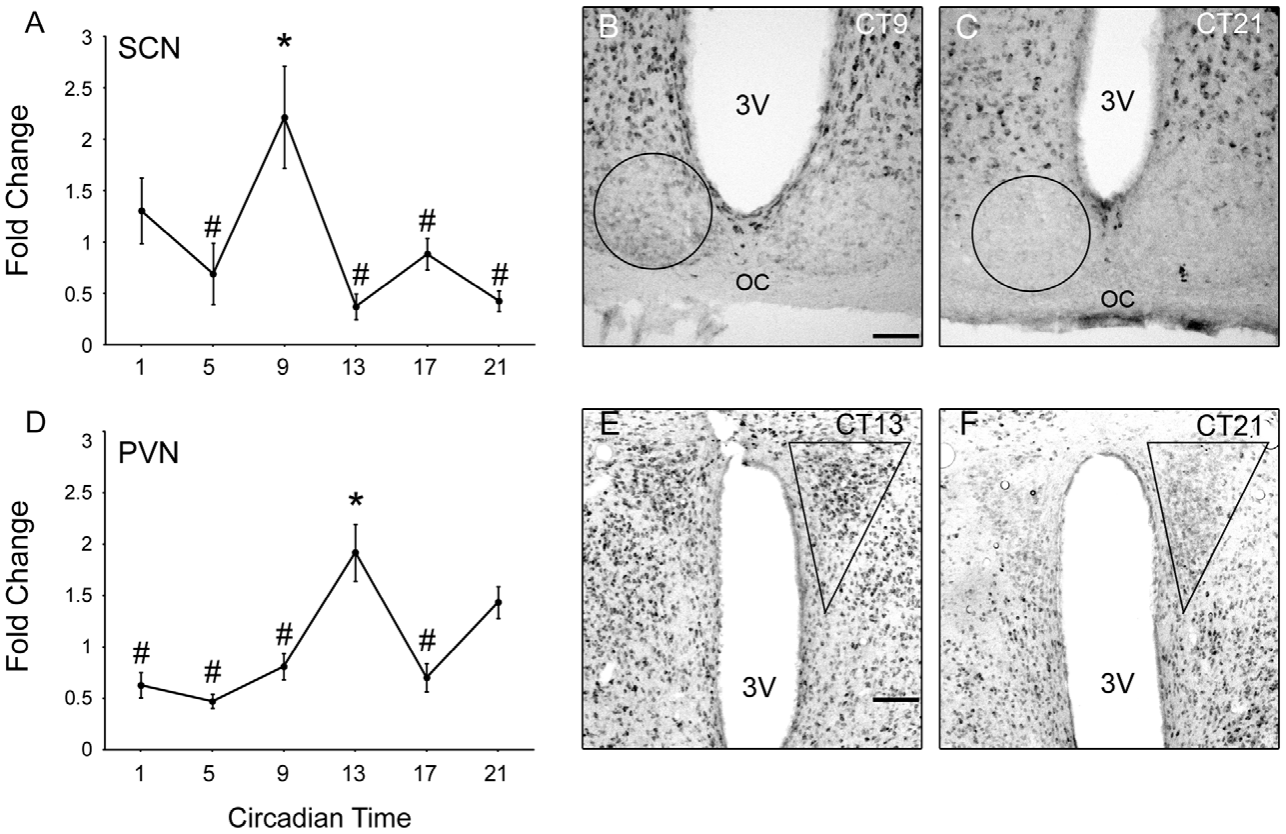
Runx2 protein immunoreactivity is rhythmic in the SCN and PVN of wild type mice. Fold change (mean ± SEM) in Runx2 protein levels measured by immunodensity analyses in the SCN (A) and PVN (D) of mice housed in 24 h DD. Data are expressed as the fold change (mean ± SEM) relative to the overall mean across times of day for all animals for each brain region. . Significant peaks levels of expression (*) compared to nadir points (#; *p*<0.05). Representative images of sections through the SCN (B and C) and PVN (E and F) at peak (B and E) and nadir (C and F) time points. Traces represent area of analyses. 3V: third ventricle; och: optic chiasm. Scale bar: 100 µm.

Next, immunohistochemistry was used to determine whether RUNX2 protein, like its mRNA, was rhythmic in the SCN. In the SCN, there was a significant effect of time of day on levels of RUNX2 immunoreactivity (F (5, 43) = 17.563, *p* = 0.042; Figure 6), under DD conditions. Optical density levels were significantly greater at CT9 than at any time assessed during subjective night (CT9 vs. CT13, 17 and 21, *p* = 0.009, 0.032 and 0.003). The time of greatest optical density of RUNX2 immunoreactivity corresponded to ∼4 h following peak *Runx2* gene expression levels in the SCN of animals housed in either LD or DD conditions (Figure 4). Overall protein concentration of RUNX2 in the SCN at the peak and nadir times of immunoreactivity density were also found to be significantly different as determined by western blot analyses (CT9 vs. CT21, *p* = 0.023).

Rhythms in the optical density of RUNX2 immunostaining were also observed in the PVN (Figure 6). In the PVN (F (5, 31) = 11.019, *p* = <0.01), peaks in optical density were seen in the early and late night compared to all times during the subjective day (CT13 vs. CT1, 5, 9, and 1, *p* = 0.002, 0.0006, 0.0056 and 0.0069; Figure 6). As in the SCN, the initial peak in optical density of RUNX2 immunostaining in the PVN occurred ∼4 h following the observed peak in *Runx2* gene expression (Figure 5).

### Rhythmic expression of *Runx2* is absent in the SCN of *Bmal1* knockout mice

The similarity in phase observed between *Runx2* and *Per1/2* gene expression rhythms suggested that *Runx2* may be a clock-controlled gene in neurons. Therefore, to determine whether rhythmic *Runx2* expression was dependent on a functional molecular clock system, gene expression within the SCN of mice lacking one or both functional *Bmal1* alleles were assayed at ZT/CT5 and ZT/CT17. Specifically, animals were transferred into DD at ZT12 and sacrificed at 17 h and 29 h later, reflecting ZT/CT5 and ZT/CT17 in the following circadian cycle. True circadian time cannot be established in these animals because their behavior becomes arrhythmic in the absence of a daily light cue. Since photic stimulation of the SCN may itself stimulate expression of *Runx2*, animals were sampled within the first day of DD without photic stimulation in effort to sample tissues prior to the breakdown of entrained rhythms. The times selected represent times of peak and nadir levels of expression of *Runx2* and clock genes as observed in WT animals (Figure 4).

**Figure 7:**
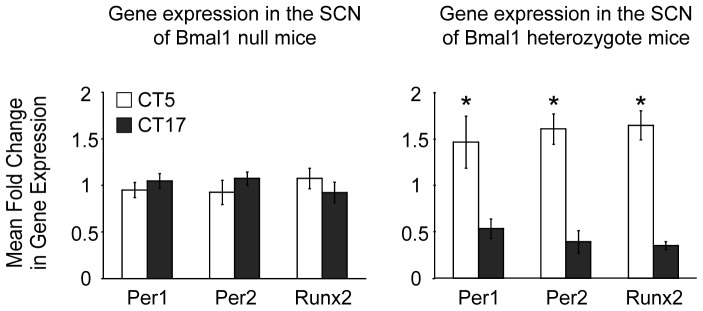
Diurnal changes in Runx2 gene expression are absent in *Bmal1^−/−^* but not *Bmal1^+/−^* mouse SCN. Fold changes in mRNA levels (± SEM) of Per1, Per2, Runx2 and Runx1 determined by real-time qRT-PCR under DD conditions. Values represent the level of normalized gene expression relative to the mean overall normalized gene expression at observed times of day. *: significant difference in levels of gene expression between times of day; *p*<0.05.

In homozygote *Bmal1^−/−^* mice, the time of day difference in *Runx2* gene expression was completely absent (CT5 vs. CT17, *p* = 0.147; Figure 7). As expected, time of day differences in gene expression of *Per1* and *Per2* were also absent in the SCN of *Bmal1^−/−^* mice (*p* = 0.388 and 0.867, respectively; Figure 6). In contrast, time of day differences in fold changes in gene expression of *Per1*, *Per2* and *Bmal1* observed in heterozygote *Bmal1^+/−^* mice mirrored those observed in WT animals [20]. Similar to previous reports, expression of both *Period* genes, *Per1* and *Per2*, were significantly greater at ZT5 than ZT17 (*p* = 0.012 and 0.00001, respectively) and the expression of *Runx2* was observed to be greater at ZT5 compared to ZT17 (*p* = 0.0007) in the SCN of *Bmal1^+/−^* mice (Figure 7) [20]. Together, these findings suggest that the circadian rhythms in *Runx2* gene expression within the mouse SCN are dependent on expression of a functional *Bmal1* gene.

### Circadian rhythms of mPer2^Luc^-mediated bioluminescence in isolated SCN from E19/20 *Runx2^+/+^*, *Runx2^+/−^* and *Runx2^−/−^* mice

In order to determine whether a complete absence of Runx2 alters SCN circadian clock gene rhythms, *Runx2^+/−^* mice homozygous for the *mPer2^luc^* allele were generated in order to measure molecular rhythms using real-time monitoring of *in vitro* bioluminescence from the SCN. Tissues were isolated on embryonic day 19–20 (E19/20) thus circumventing the neonatal lethality of the homozygote *Runx2^−/−^* genotype [21].

**Figure 8:**
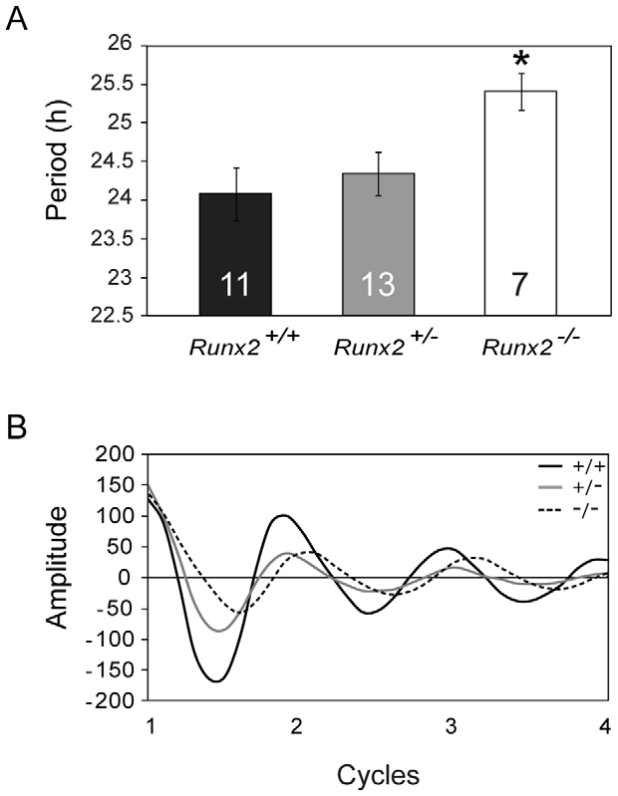
Mean period of mPer2^Luc^-mediated bioluminescence rhythms is lengthened in the SCN of *Runx2* deficient mice. A) Mean period (± SEM) of mPer2^Luc^ rhythms in SCN cultures of *Runx2^+/+^* (black), *Runx2^+/−^* (grey) and *Runx2^−/−^* (white) determined from real-time monitoring of bioluminescence from isolated E19/20 mice. Inlayed number represents n value of experimental group. B) Representative trace of mPer2^Luc^-mediated bioluminescence observed over several cycles from *Runx2^+/+^* (black), *Runx2^+/−^* (grey) and *Runx2^−/−^* (dashed) SCN cultures. *: significant difference in levels of gene expression between times of day; *p*<0.05.

Overall, there was a significant difference in the mean period length of mPer2^Luc^ rhythms in SCN cultures from *Runx2^+/+^*, *Runx2^+/−^* and *Runx2^−/−^* mice (F (2, 28) = 3.537, *p* = 0.043; Figure 8). Post hoc analyses confirmed that the mean period of the mPer2^Luc^ rhythms in the SCN of *Runx2^−/−^* E19/20 mice (n = 7, 25.3±0.26 h) was significantly longer than those observed in the SCN of either *Runx2^+/−^* (n = 13, 24.20±0.26 h, *p* = 0.039) or *Runx2^+/+^* (n = 11, 23.92±0.34 h, *p* = 0.047) littermates (Figure 8A and B). No significant difference in the mean period of the mPer2^Luc^ rhythms was seen between *Runx2^+/−^* and *Runx2^+/+^* littermates (*p* = 0.328). We did not observe a difference in the mean amplitude of bioluminescence levels between genotypes (*p*>0.1). There were no significant differences in any of the amplitude parameters, but this may be partially due to variability between cultured samples. Moreover, in liver tissue which does not express *Runx2*, there was no difference in the mean period of bioluminescence between genotypes (data not shown; *p* = 0.473).

### *Runx2^+/−^* mice display lengthened period of free-running activity

To determine whether *Runx2* deficiency leads to *in vivo* consequences in circadian behavior, the wheel-running rhythms of *Runx2^+/−^* mice were compared to those of *Runx2^+/+^* mice. As noted above, heterozygote animals were used since *Runx2*-null mice are postnatal lethal [21].

**Figure 9:**
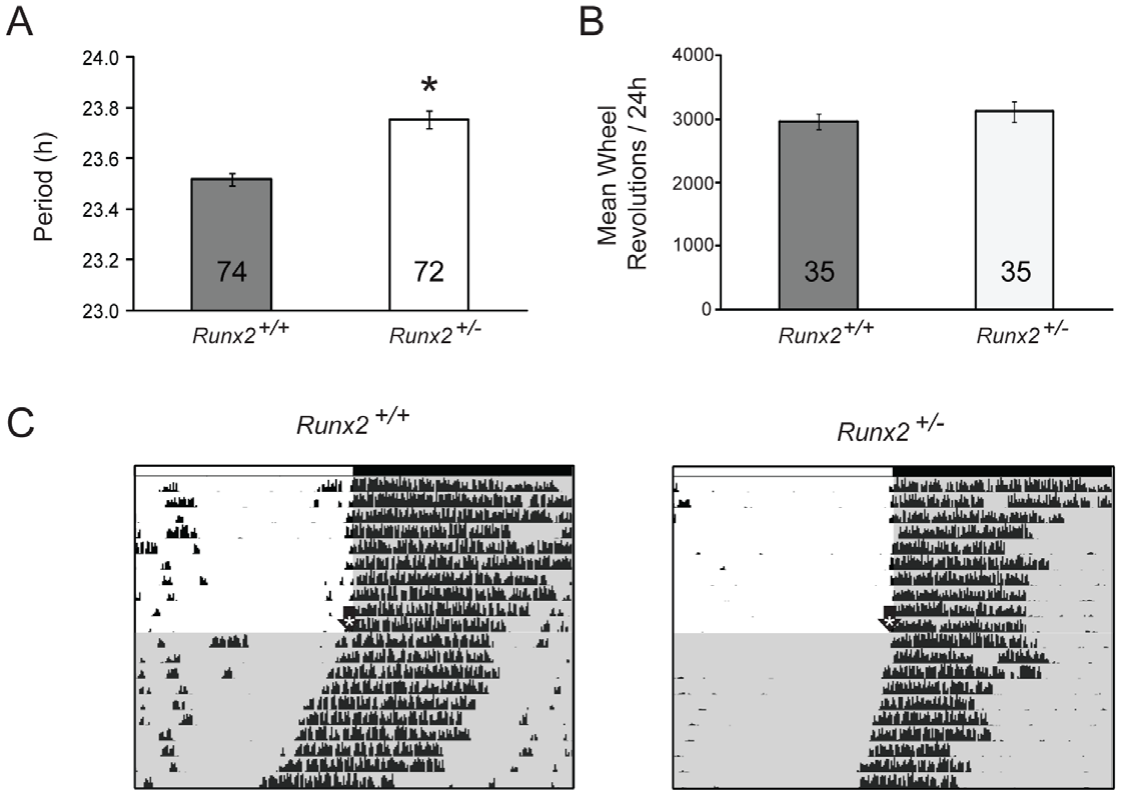
Free-running period of running wheel activity is lengthened in *Runx2^+/−^* mice. A) Mean (± SEM) free-running period of *Runx2^+/+^* (grey) and *Runx2^+/−^* (white) mice. Inlayed number represents n value of experimental group. B) Mean ± SEM wheel revolutions of male *Runx2^+/+^* (grey) and male *Runx2^+/−^* (white) entrained to a 12∶12 h LD schedule. Inlayed number represents n value of experimental group. C) Representative running wheel activity records (upper panels) for *Runx2^+/+^* (left panels) and *Runx2^+/−^* (right panels) mice. Activity records are in single-plotted format with each horizontal line represents a 24 h period. In 12 h∶12 h LD conditions, time of day is represented by white and black horizontal bars indicating periods of day and night, respectively, with dark onset marking ZT12. Black arrow with white asterisk: transition into 24 h DD. *: significant difference between genotypes; *p*<0.05.

In free-running conditions, *Runx2^+/−^* mice displayed a significantly longer mean free-running period (n = 72, 23.78±0.03 h) than that of *Runx2^+/+^* males (n = 74, 23.54±0.04 h, *p* = <0.001) (Figure 9A and C). The mean total wheel revolutions per day were not significantly different between *Runx2^+/+^* (2959.73±126.40) and *Runx2^+/−^* mice (3120.60±166.68, *p* = 0.126; Figure 9B), indicating that differences in free-running period are not due to decreased overall locomotor activity.

Exposure to environmental cues, such as light, can induce phase-dependent shifts in activity rhythms. To investigate whether Runx2 plays a role in photic resetting of the clock, *Runx2^+/+^* and *Runx2^+/−^* mice were subjected to phase shifting light pulses during either subjective day, or early or late subjective night. As expected, running wheel activity was significantly shifted following exposure to a light pulse during late (CT21 vs. no pulse; *p* = 0.000003) or early subjective night (CT 15 vs. no pulse; *p* = 0.0005) but not to light exposure during subjective day (CT6 vs. no pulse; *p* = 0.704; Table 1). Genotype comparisons revealed that direction and amplitude of phase shifts in response to light pulses at these different circadian times did not differ between *Runx2^+/−^* and *Runx2^+/+^* littermates (CT6 pulse, *p* = 0.566; CT15 pulse, *p* = 0.975; CT21 pulse, *p* = 0.159; Table 1).

**Table 1:** *Runx2^+/−^* mice display responses to phase shifting light pulses indistinguishable from *Runx2^+/+^* mice.

Genotype	Light Pulse at CT6	Light Pulse at CT15	Light Pulse at CT21
Runx2^+/+^ (n = 6)	−0.140±0.491 h	−1.136±0.378 h	2.362±0.667 h
Runx2^+/−^ (n = 6)	0.270±0.377 h	−0.933±0.209 h	2.088±0.578 h

Mean phase shifts (h ± SEM) for wild-type and *Runx2^+/−^* mice exposed to a light pulse at CT6, CT15 or CT21. See text for *p* values.

## Discussion

We found that *Runx2* mRNA and protein expression is rhythmic and under the control of circadian clock network in the SCN, as well as in several other brain regions previously shown to exhibit rhythmic clock gene expression [36], [37]. In addition, Runx2 appears to play a functional role in the regulation of cellular and behavioral rhythms, with lengthened free-running period being observed in Runx2 mutant SCN tissues and animals.

In the SCN, the phase of peak *Runx2* expression was similar to that of *Per1* and *Per2* suggesting the possibility that *Runx2*, like *Per1* and *Per2*, may be a target for Bmal1/Clock transactivation. Indeed, in the absence of a functional *Bmal1* gene product, a rhythm in *Runx2* mRNA in the SCN of mice housed under DD conditions was undetectable, again mirroring the effects on *Per1* and *Per2*. This effect of the *Bmal1* knockout on *Runx2* gene expression suggests that *Runx2* is a clock-controlled gene dependent on *Bmal1* activity. Consistent with this, a putative Bmal1-response element (E-box) has been described in the region upstream of the *Runx2* promoter, although the functionality of this site remains to be validated [38]. A comprehensive study of the promoter regions of Runx2 would provide greater insight into the possible mechanisms of Bmal1-mediated *Runx2* expression [39].

Alternatively, rhythmic expression of the *Runx2* gene may not be directly mediated by *Bmal1* but rather under the transcription control of one or several other CCGs. One such CCG, known to be rhythmically expressed in the SCN, is *transforming growth factor ß* (*TGF-ß*) [40]–[42]. In osseous tissues, *Runx2* expression and activity is repressed by the signalling cascade activated by TGF-ß [41], [42]. Since *TGF-β* expression and signalling activity are elevated during the subjective night in the SCN, a time when *Runx2* expression is at its lowest, TGF-β may be having a similar repressive effect on the *Runx2* transcription in the SCN [40]. Given the evidence for the non-photic phase shifting properties of TGF-ß signalling in peripheral tissues [43], it is interesting to speculate its capacity to do so in the SCN is attributable to its effects on *Runx2*.

The results of the *in vitro* and *in vivo* experiments reported here suggest that Runx2 participates in regulating circadian period length through its influence on molecular and/or signalling pathways occurring at the level of the SCN. Within the SCN and other tissues, period length is largely due to the timing of intracellular molecular rhythms [44], and there are several possible mechanisms by which Runx2 may act to lengthen the period of cellular rhythms. First, the timing of the intracellular rhythms may be modified by actions of Runx2 on transcription of clock genes or their regulators leading to a lengthened period of the individual cellular oscillators comprising the SCN. The runt-domain of the Runx2 protein binds DNA in a site-specific manner by recognizing sequences identified as of 5′-puACCpuCA-3′ [45]. A cursory analysis identified a number of putative Runx2 binding sites within the sequences of clock genes including *Bmal1* and *Per2* (NCBI reference sequence: AC_000029.1, NC_000067). Further studies are needed to identify functional Runx2 binding sites in clock gene sequences and to determine the influence of Runx2 on the expression of these genes. Runx2 also contains a number of protein-protein interaction domains that act to recruit proteins to downstream gene targets, including transcription activators (i.e. p300, CCAAT/enhancer binding proteins) and repressors (i.e. HDACs) [46]–[48]. The diversity of the multi-protein complexes formed through Runx2-mediated scaffolding is further increased by a number of potential post-transcriptional modifications to Runx2 [49], [50]. Thus, in addition to acting directly through its DNA-binding sequences, Runx2 may act indirectly to regulate transcription of clock genes and/or CCGs by facilitating recruitment of other factors that, in turn, serve to facilitate or repress target genes.

The period lengthening effect of *Runx2^+/−^* observed *in vivo* was also observed in the period of the bioluminescence rhythms detected from cultured SCN tissues collected from *Runx2^−/−^* mice. Unlike activity rhythms measured *in vivo*, no significant change in the period length was detected in *Runx2^+/−^* SCN cultures. However, when the mean period length value of *in vitro* bioluminescence rhythms measure from the SCN tissues of *Runx2^+/−^* mice is compared to the mean period of free-running rhythms of Runx2^+/−^ mice, both show a similar magnitude of period lengthening (16.8 min *in vitro* vs.14.4 min *in vivo*). The lack of statistical significance between the periods of molecular rhythms measured from *Runx2^+/+^* and *Runx2^+/−^* SCN tissues therefore likely lies in the variability of the measured bioluminescence rhythms *in vitro*. This variability could be due to a number of factors including a relatively smaller sample number for each group under *in vitro* conditions versus the *in vivo* sample sizes or the discrepancies in period lengths measured from tissue cultures and behavioral parameters [51]–[54]. This latter phenomenon was observed in the animals harboring the mutations in the circadian clock factors, Clock and CK1ε [54], [55]. Previous studies correlating period in behavioral rhythms *in vivo* to molecular rhythms *in vitro* have examined animals harboring mutations with dramatic differences in period length [56]–[58] and under conditions where both gene alleles are compromised. Another factor may have been the differences in age of the mice in the *in vivo* (adult) and *in vitro* experiments (embryonic). Thus it is possible that the effects we observed in haplodeficient animals are only manifested in adulthood perhaps due to compensatory mechanism(s) during development that either wane or are insufficient later in life.

Beyond the SCN, detectible expression levels of *Runx2* were rhythmic in the PVN, OB and hippocampus. The phase of the *Runx2* rhythm was shifted by ∼4 h in each of these brain regions under DD conditions compared to its phase in the SCN. This phase lag may be of physiological importance since the synchronization of oscillators to one another may be critical in coordinating neural activity and responses to external cues (i.e. light, food). Such a phase lag (3–9 h) relative to their maximal expression in the SCN is typical of genes expressed in peripheral tissues whose circadian oscillations are driven and/or synchronized by a signal led by the circadian pacemaker [22], [59]. Support for SCN-influenced expression of *Runx2* in these peripheral tissues can be derived from the observation that shifts in the phase of *Runx2* oscillations were similar to the lag observed for the circadian clock gene, *Per2*. Whether the oscillations of *Runx2* in these brain regions are driven and/or synchronized by the SCN remain to be elucidated. It may be that the rhythmic expression of *Runx2* in the PVN, a target of SCN efferents, is driven by the SCN and would become arrhythmic in its absence [36], [60]–[63]. In contrast, the OB has been convincingly shown to contain independent, self-sustained oscillators that do not rely on the SCN [64], [65]. Since rhythms in the OB are synchronized rather than driven by the SCN, oscillations in *Runx2* in the OB would likely persist in SCN-lesioned animals [65]. Rhythmic expression of clock genes, including *Bmal1* and *Per2*, has previously been described within the HP; however, the dependency of these rhythms on the SCN has not been thoroughly evaluated [37], [66]. Finally, Runx2 protein was found to expressed in numerous other areas within the brain, as are clock genes suggesting that Runx2 may influence circadian modulation of a variety of physiological and behavioral functions [24], [37], [66].

Runx2 deficiency is known to have clinical consequences in skeletal development and bone homeostasis. Cleidocranial dysplasia (Online Mendelian Inheritance in Man # 119600) is a rare genetic disorder resulting from an autosomal dominant mutation(s) or complete loss of a functional Runx2 gene copy [67]. In general, patients with this disorder have abnormal osteogenic development and present with a number of skeletal and dental deformities which vary in severity depending on the causal gene mutation [68]. To date, no circadian or sleep disorders have been linked with CCD. Given our findings on the effects of Runx2 on period length, it would be worthwhile to assess the status of circadian biomarkers and sleep patterns in individuals with CCD to determine if the effects of *Runx2* haplodeficiency we observed in mice translate to humans.

In summary, *Runx2* appears to be a circadian clock-controlled gene that is expressed rhythmically in the SCN and other regions of the murine brain. This expression occurs in a circadian fashion with greatest levels of expression occurring during the subjective day and this rhythm is dependent on the expression of *Bmal1* suggesting that *Runx2* is a circadian controlled gene. Decreased *Runx2* gene expression leads to significant lengthening of the period of behavioral rhythms *in vivo* and lengthened *mPer2^Luc^* rhythms *in vitro*. Future studies will be needed to identify the precise mechanisms by which *Runx2* is regulated by circadian clock genes within the SCN as well as in bone and other peripheral tissues, and its potential impact on other clock-controlled genes.
